# Rare and novel variant load threshold for *KIF7*, *GJA1* and *PDE1C* genes elevates the risk of severity of congenital heart defects in Down syndrome

**DOI:** 10.1371/journal.pone.0326566

**Published:** 2025-06-26

**Authors:** Agnish Ganguly, Samudra Pal, Srilagna Chatterjee, Madhusudan Das, Sumantra Sarkar, Sujay Ghosh

**Affiliations:** 1 Cytogenetics-Genomics and Down syndrome Research Unit, Department of Zoology, University of Calcutta, Kolkata, West Bengal, India; 2 Department of Biotechnology, School of Life Sciences, Swami Vivekananda University, Barrackpore, West Bengal, India; 3 Department of Zoology, University of Calcutta, Kolkata, West Bengal, India; 4 Department of Pediatric Medicine, Diamond Harbour Government Medical College & Hospital, Diamond Harbour, West Bengal, India; Mansoura University Faculty of Medicine, EGYPT

## Abstract

Individuals with Down syndrome (DS) exhibit a higher incidence of congenital heart defects (CHD). The objective of the present study was to investigate ethnicity-specific genetic variants that increase the risk of CHD in children with DS from the Indian Bengali population. We conducted whole exome sequencing of the genomes of Down syndrome children with and without CHD and subsequently tested the identified variants in a larger cohort (N = 1798). Our findings revealed two rare variants, *KIF7* rs138354681 and *GJA1* rs778110855, as well as one novel variant, *PDE1C* PP785745, present in children with DS and CHD but absent in those without CHD. In-silico analyses indicated that these variants are pathogenic. The frequencies of the heterozygous genotypes for *KIF7* rs138354681, *GJA1* rs778110855, and *PDE1C* PP785745 were recorded as 0.027, 0.016, and 0.032, respectively. Among the 31 carriers identified, 18 individuals exhibited two variants, while four were found to have three co-occurring variants. The majority of these individuals required surgical intervention for correction, in contrast to single variant carriers, of whom only three out of nine needed surgeries. A polygenic risk score analysis revealed higher score to be significantly associated with both the presence of multiple variants and the subsequent need for surgical correction. We hypothesise that the synergistic effects of multiple variants heighten the severity of CHD, particularly in cases of ventricular septal defects, thereby necessitating surgical correction. These findings significantly enhance our understanding of the unique population-specific aetiology of CHD and the basis for the severity of its clinical presentation in individuals with Down syndrome.

## Introduction

Trisomy 21 (T21) or Down syndrome (DS) is the most common aneuploidy that completes full term and is characterised by nearly eighty co-occurring conditions. Among the live-born individuals with DS, nearly 50% suffer from congenital heart defects (CHD) [1]. CHDs pose serious health challenge, being the largest contributor to DS infant mortality, especially within the first 2 years of life. Globally, the most prevalent congenital heart defect (CHD) phenotypes observed among individuals with DS are atrioventricular septal defects (AVSDs), followed by ventricular septal defects (VSDs), tetralogy of Fallot (TOF), and atrial septal defects (ASDs), which may vary across ethnicity [2].

The increased prevalence of CHDs in the DS population is strongly attributed to T21 associated genomic dysregulation during foetal development that markedly elevates the susceptibility to this disorder. But it does not provide absolute answer as some of the DS individuals do not carry CHD and there are considerable variations in clinical presentation and severity in different forms of CHDs, too. It is plausible that diverse allelic variations of multiple genes across the genome, complex cross talking among them with a T21 genetic backdrop contribute to variable presentation of CHD associated with DS. Efforts to elucidate the genetic underpinnings of CHDs in DS are particularly valuable, as they not only enhance our understanding of the genetic etiology in DS but also shed light on the underlying causes of CHDs in non-DS cases and the fundamental mechanisms of human cardiogenesis.

Multiple studies have looked into the effect of common genetic variants in CHDs associated with DS, including the most extensive genome-wide association study (GWAS) conducted to date. This study encompassed 210 cases of complete AVSD in individuals with DS and 242 controls with DS exhibiting structurally normal hearts [3]. Despite the ample sample sizes enabling the detection of common variants with substantial effect sizes, no common variants, whether single nucleotide polymorphisms (SNPs) or copy number variants (CNVs), reached genome-wide significance in these investigations [4,5]. This observation suggests that large-effect common variants likely do not play a substantial role in the development of CHDs in individuals with DS. However, it is conceivable that common variants with low to moderate effects, including SNPs and structural variants, may contribute to the risk, potentially operating in a cumulative manner [6].

Besides, studies focussed on rare variants associated with atrioventricular septal defect have identified some polymorphisms that associate CHD, both in individuals with DS and those with non-syndromic AVSD. In a targeted sequencing investigation involving 26 candidate genes for AVSD, conducted on 141 cases of DS with AVSD and 141 DS controls with structurally normal hearts, rare variants of the genes from the vascular endothelial growth factor (VEGF) pathway, were found associated with CHD [7]. Another comprehensive copy number variant (CNV) analysis performed on 210 cases of DS with AVSD and 242 DS controls with structurally normal hearts revealed a suggestive enrichment of large rare deletions among the ciliome regulatory genes from DS case subjects, and it provides additional insights into the potential involvement of rare variants in CHD development [4].

A subsequent study, involving 198 DS with CHD cases and 211 DS with normal heart, explored CNVs on the triplicated chromosome 21, reported that among African-Americans, controls exhibited more bases covered by rare deletions compared to African-American cases. Conversely, among Caucasians, cases exhibited a higher number of genes intersected by rare duplications in comparison to Caucasian controls [5]. This underscores the importance of considering ethnic diversity in understanding the impact of rare variants on CHD in DS.

Studies on Indian population for understanding the genetic etiology of CHD in DS is very limited. Few studies have looked into the association of polymorphisms of folate metabolism pathway genes with DS associated CHD. One study reported A66G, C524T, T19775C and 19778_19778delG polymorphisms in the *MTRR* gene as risks for CHD in DS [8], while another study reported *MTHFR* C677T in addition to the *MTRR* A66G polymorphism elevates risk of CHD in DS [9]. Our group previously reported *SLC19A1* A80G polymorphism alone, and in concert with *MTHFR* C677T, exacerbates the risk of AVSD in DS individuals [10]. Beside folate regulators, two published works reported association of polymorphisms of *CRELD1* gene with DS linked CHD in Indian population. The first of two was from our team in which we [11] reported an association of *CRELD1* polymorphic haplotypes (rs9878047-rs3774207-rs73118372) with increased odds in favour of AVSD among DS individuals and, second one by Asim et al., 2018 [12], who reported a novel missense VCV001686602.1 variant in *CRELD1* gene, to be exclusively present among individuals with DS and AVSD.

In the current study, we have aimed to identify the population-specific rare genetic variants that might contribute to the elevated incidence of CHD in DS individuals in Bengali speaking Indian cohort from West Bengal, otherwise known as the Indian Bengali population. Additionally, for the first time ever, we tried to elucidate the genetic underpinning of variation in severity CHD in DS. We designed our study to find out the answer of two questions: 1) is there any population specific unique rare genetic variants that increase risk of CHDs in DS from India? 2) Is there a difference in the genetic basis of CHDs in DS that require surgical intervention and those that do not?

## Materials and methods

The study was conducted in accordance with the ethical criteria of the Declaration of Helsinki and the research regulations specified by the Indian Council of Medical Research (ICMR). The ethics committees established by the University of Calcutta reviewed the methodologies and approved the study (Approval No.: CU/BIOETHICS/HUMAN/2306/ 3044/2018).

### Study cohort

A total of 1319 Down syndrome participants, classified as, 677 free and complete T21 (2n = 47, + 21, XX or XY) individuals with CHD (we will refer this group as T21 + CHD) and 642 free and complete T21 (2n = 47, + 21, XX or XY) individuals without CHD (we will refer this group as T21-CHD) were recruited. Another 479 euploid healthy individuals (referred as D21, here on) were also recruited. All participant recruitment was done randomly from different areas of the state of West Bengal, India, between 15^th^ May 2018 and 30^th^ October 2023. It was ensured that the T21 and D21 participants were demographically matched, to avoid any discrepancy. Participants were primarily from Kolkata, followed by Diamond Harbour (southern part of the state), Malda and Siliguri (northern part of the state). The CHD status of the T21 + CHD group individuals was confirmed through chest X-rays, electrocardiographs and ultrasonic echocardiograms. Written informed consent was obtained from each participant and/ or their parents or legally appointed representatives, as applicable. Participating families were interviewed in person for epidemiological data collection. The data were anonymized and strict confidentiality was maintained. In this study we considered T21 + CHD as case and T21-CHD and euploids as control groups. Case and control subjects were all age, sex and height matched. We were careful to select case and control subjects from the same locality to maintain maximum demographic similarity among them. Additionally, we have taken care for judicious selection of the T21 + CHD and T21-CHD subjects from the similar socio-economic strata to negate any possibility of sampling bias.

### Tissue collection and DNA isolation

About 3 ml of peripheral blood samples were collected in EDTA-coated vacutainers from 1798 participating individuals by expert clinician collaborators. Isolation of genomic DNA from whole blood samples was performed using QIAamp Blood Mini Kit (QIAGEN, Hilden, Germany) adhering to manufacturer’s protocol.

### Trisomy 21 status confirmation

We confirmed the free and complete trisomy21 (T21) status of all the T21 subjects by classical karyotyping and by STR-genotyping using panel of Hsa21q specific microsatellite markers spanning from centromere to telomere (S1 Fig). Additionally, peri-centromeric markers were used to interpret parent of origin of supernumerary Hsa21. All the cases we analysed were of maternal meiosis I nondisjunction cases.

### Whole exome sequencing and data analysis

The experimental workflow for whole exome sequencing (WES) was conducted at MedGenome Labs Ltd., located in Bangalore, Karnataka, India (https://diagnostics.medgenome.com/whole-exome-analysis/). Exonic regions were enriched using the Agilent SureSelect V4 kit (Agilent Technologies, CA, USA) and sequenced on an Illumina NOVASeq 6000 platform (Illumina, CA, USA) with 101-base pair (bp) paired-end reads. The overall alignment rate of the samples to the human reference genome (hg38) was approximately 99.96%. Post-alignment analysis was carried out using the TWIST_EXOME_REFSEQ panel (36,715,240 bp), which covers 26,642 genes. The average coverage of the samples across the panel was approximately 98.35%. The open-source tool fastq-mcf was employed to detect and remove sequencing adapters, primers, and low-quality nucleotides at the ends of the reads. Alignment of the adapter-trimmed FASTQ files to the human reference genome (hg38) was performed using Sentieon’s version of BWA. Subsequently, the alignment SAM files were sorted and converted to binary compressed BAM files using Samtools. To recalibrate the quality scores of all reads in the BAM files, Sentieon’s version of the GATK BaseRecalibrator was applied. Variant calling was performed using Sentieon’s GATK HaplotypeCaller and UnifiedGenotyper, generating results in VCF format. Variants were annotated using the in-house pipeline, VariMAT (Variation and Mutation Annotation Toolkit). The entire pipeline was further validated using CLC Workbench version 23.0 (CLCbio; Qiagen Bioinformatics, Aarhus, Denmark).

### Bi-directional sanger sequencing

We performed Sanger sequencing to validate the selected variants in the larger sample size of 677 T21 + CHD, 642 T21-CHD and 479 D21 individuals. The exon 12 of *KIF7*, exon 2 of *GJA1* and exon 3 of *PDE1C* were amplified using polymerase chain reaction (PCR). Primers were designed using Primer3 (v4.1.0) and the OligoAnalyzer tool (https://eu.idtdna.com/) from Integrated DNA Technology was employed for primer testing. The dideoxy sequencing primer sets used for the respective gene are: *KIF7* exon 12- F: 5’-TCTATCTGCCTGCCCTTGTT-3’/ R: 5’-CCGCCAGCTCAGCCTATT-3’; *GJA1* exon 2- F: 5’-GAGCCCTGCCAAAGACTGT-3’/ R: 5’- TCATGTCCAGCAGCTAGTTT-3’; *PDE1C* exon 3- F: 5’-CCTGCTCCTTTGGTCCCATA-3’/ R: 5’-AGATGAGGCGGTACAGAGAG-3’. The PCR reaction was conducted in a 30 μl volume using 50–100 ng of DNA, 1 μl of primers (10 mmol/L), 0.2 μl of dNTPs (10 mmol/L; Invitrogen Carlsbad, CA, USA), 1.5 μl of MgCl2 (50 mmol/L), 1 x PCR buffer, and 0.8 μl of Taq Pol (5 units/1 μl; Invitrogen, California, USA). All genotyping procedures, i.e., WES and bidirectional Sangers sequencing were accomplished blinded without knowing the CHD status of the subjects.

### In-silico prediction analysis

We used multiple software tools to predict the potential damaging effects of the studied variants on the respective proteins. For each variant, the mutated sequences and/or altered amino acids were ‘*input*’ according to the required formats of the respective programs, and the resulting ‘*outputs*’ were analyzed. The PolyPhen-2 program [13] (http://genetics.bwh.harvard.edu/pph2/) was used to qualitatively categorize the variants as ‘benign’, ‘possibly damaging’, or ‘probably damaging’, based on FPR thresholds that are specifically optimized for different models, such as HumDiv and HumVar. We used the MutationTaster [14,15] (https://www.mutationtaster.org/) software to estimate the probability of a mutation being either deleterious or benign. The prediction is supported with a probability score that the reflects the confidence of the prediction. The ‘Deleterious’ effect of the variants was also predicted using the PROVEAN [16] (http://provean.jcvi.org/index.php) program. A PROVEAN score at or below the threshold of −2.5 is predicted as ‘deleterious’, while scores above this threshold predict a ‘neutral’ effect. To check whether our variants in question were ‘Tolerated’ or not, we used the SIFT (Sorting Intolerant from Tolerant) [17] (https://sift.bii.a-star.edu.sg/) program. A SIFT score between 0 to 0.05 suggests the ‘Intolerant’ nature of the variant whereas, one between 0.05 to 1, predicts it to be tolerated. We also used Combined Annotation Dependent Depletion (CADD) [18] (https://cadd.bihealth.org/) tool to score the deleteriousness of the variants. The deleterious effect of the variants at the 10th-% of CADD scores are assigned to CADD-10, top 1% to CADD-20, top 0.1% to CADD-30. We further studied the interaction network of the three genes *KIF7*, *GJA1* and *PDE1C* using GeneMANIA tool in the Cytoscape software [19] and STRING program. To confirm the availability/ rarity/ frequency of the identified variants we used publicly available population databases such as the Genome Aggregation Database (gnomAD) and 1000 Genomes Database

### Statistical analyses

Polygenic Risk Scores (PRS) were calculated for each individual as a weighted sum of genotype values according to variant-specific effect sizes (β) derived from earlier reported genome-wide association studies (GWAS). We considered presence of CHD as ‘penetrance’ and needs of surgical intervention as ‘severity’ and ‘expressivity’ of the trait. The collected genotype information for three variants (*KIF7*, *GJA1*, and *PDE1C*) were represented as 0, 1, or 2, which indicates the number of risk alleles present in the T21 and D21 subjects. The data presented as binary phenotype variables (For subject needed surgery: 1 = cases, 0 = controls). Polygenic risk scores (PRS) for those who were assigned to the surgery and non-surgery groups were compared. The distribution of the PRS scores was measured with a Shapiro-Wilk test that revealed the absence of a normal distribution. Accordingly, non-parametric statistical approaches were applied for the subsequent analysis.

In order to compare the PRS scores of the surgery and non-surgery groups, a Mann-Whitney U test was conducted. The effect size was approximated with rank-biserial correlation (r). Another Spearman’s rank correlation test was also conducted in order to assess the association between PRS scores and surgery status.

Data visualization also involved a box plot to represent PRS score distributions between groups and a histogram with kernel density estimation (KDE) for presenting overall PRS score distribution. Statistical analyses and data visualization were carried out using Python (SciPy, Seaborn, and Matplotlib libraries) and R version 4.4.3. The PRS was estimated as: PRS = (Genotype *KIF7* × β*KIF7*) + (Genotype *GJA1* × β*GJA1*) + (Genotype *PDE1C*×β*PDE1C*) where β represents the effect size for each SNP. PRS calculation and statistical analyses were performed in R (version 4.4.3 & PLINK version 1.9).

## Results

A total of 1319 T21 individuals were recruited in this study, of which 699 were male and 620 were female. The age range was between 1 year 7 months and 13 years, with a mean age of 7.55 years. Out of the 1319 T21 individuals, 677 individuals (51.3%) were diagnosed with congenital heart defects. Among them, 309 (45.7%) were male and 368 (54.3%) were female. The most frequent congenital cardiac defect recorded in our study cohort is ventricular septal defect (T21 + VSD) with a prevalence of 33.7% (228/677). This was followed by atrial septal defect (T21 + ASD) at 25.7% (174/677), atrioventricular septal defect (T21 + AVSD) at 22.3% (151/677), patent ductus arteriosus (PDA) (T21 + PDA) at 10.9% (74/677), patent foramen ovale (PFO) (T21 + PFO) at 6.2% (42/677) and tetralogy of Fallot (T21 + TOF) at 1.2% (8/677). Among the 479 healthy D21 individuals, 254 were male and 225 were female. The age range of the D21 group was between 2 year 6 months and 12 years and 3 months, with a mean age of 7.35 years. None of them carried any CHDs.

The WES primary yield exhibited each subject carried approximately 26,500 genetic variants. We did not consider any synonymous variants in this study. Remaining variants that were reported in genes associated with cardiac development were filtered. Among them, potentially pathogenic variants that were present in at least 3 T21 + CHD individuals in the WES analysis, were further considered. Two rare variants and one novel variant were identified after this filtering, and these are *KIF7* (Gene ID: 374654; cytogenetic location:15q26.1; OMIM# 611254) rs138354681, *GJA1* (Gene ID: 2697; cytogenetic location: 6q22.31; OMIM# 121014) rs778110855 and a novel *PDE1C* (Gene ID 5137; cytogenetic location:7p14.3; OMIM# 602987) PP785745. Among these three genes, *KIF7* codes a cilia-associated protein and regulator of the sonic hedgehog (*SHH*) signalling pathway. The *GJA1* gene encodes a member of the connexin family and a key component of gap junctions of cardiomyocytes. The third gene *PDE1C* codes a member of 3’5’-cyclic nucleotide phosphodiesterase and regulate vascular smooth muscle proliferation and remodelling. We performed bi-directional Sanger sequencing to validate all three variants in 677 T21 + CHD individuals and 642 T21-CHD individuals. Interestingly, we did not find any pathogenic variant in any of the known genes from the pre-established well known Congenital Heart Disease Gene Panel (CHDGG) of Mayo clinic (https://www.mayocliniclabs.com/test-catalog/Overview/617197).

The rare variant rs138354681 (Highest MAF: 0.01) in *KIF7* exon 12 shows T > C transition that causes alteration of the 834^th^ glutamine residue to an arginine. Upon screening, the rs138354681 ‘TC’ genotype was found among 18 T21 + CHD individuals. Among the 18 T21 + CHD individuals, 14 individuals were suffering from VSD while the rest 4 reported AVSD. Among the D21 participants only 3 participants carried the ‘TC’ genotype. The frequency of the minor allele ‘C’ in the case subjects was recorded as 0.027 in the T21 + CHD group, 0.061 in the T21 + VSD group, 0.026 in the T21 + AVSD and 0.014 in the entire T21 cohort including both T21 + CHD and T21-CHD individuals, while it was recorded as 0.006 in the D21 group. None of the D21 carriers reported any cardiac defect phenotype. Upon comparing the genotype frequencies between T21 + CHD and D21 groups, employing Fisher’s exact test, it was revealed that the ‘TC’ genotype shows statistically significant (p value < 0.05) association with T21 + CHD over D21 variant carriers (p value = 0.0123; Odds Ratio = 4.334; 95% Confidence Interval = 1.269–14.801). No ‘TC’ genotypes were found among the T21-CHD group. The second variant that we reported was a rare frameshift deletion (delC), namely rs778110855 (Highest MAF < 0.01) in the 2^nd^ exon of the *GJA1* gene, present in 11 T21 + CHD individuals. The deletion was present only in the heterozygous form, i.e., ‘C/-’  among these individuals. The frequency of the minor allele (delC) in the T21 + CHD group, T21 + VSD group, and the entire study cohort are 0.016, 0.048 and 0.008, respectively. All these 11 T21 + CHD individuals were reported to have VSD. This delC frameshift mutation alters the 311^th^ alanine residue to valine, and induces a premature stop codon 37 residue downstream. None of the T21-CHD individuals or the D21 individuals carried this frameshift mutation. The third reported variant, is a novel missense transition, PP785745 in the 3^rd^ exon of the *PDE1C* gene. This G > A change alters the 66^th^ glutamic acid residue to a lysine residue in the protein. We found only the ‘GA’ heterozygous genotype among 22 T21 + CHD group individuals and 6 D21 group individuals in our study cohort. Interestingly, among these 22 T21 + CHD individuals, 16 were reported for VSD, and 6 for AVSD. The minor allele ‘A’, showed an allelic frequency of 0.032 in the T21 + CHD group, 0.07 in the T21 + VSD group, 0.04 in the T21 + AVSD group and a frequency of 0.017 in the entire T21 study cohort. We did not find any minor allele carrier in the T21-CHD group. There was no cardiac defect phenotypes reported in any of the 6 D21 carriers. A comparison of genotype frequencies between the T21 + CHD and D21 groups using Fisher’s exact test demonstrated a statistically significant association (p value < 0.05) of the ‘GA’ genotype with T21 + CHD (p value = 0.0324; Odds Ratio = 2.648; 95% Confidence Interval = 1.045–6.582).

The details of each variant and their genotypic distribution among the groups are presented in Table 1. Interestingly we did not find any of these variants among the T21 + ASD, T21 + PDA, T21 + PFO and T21 + TOF groups.

**Table 1:** Details of the three gene variants, *KIF7* rs138354681, *GJA1* rs778110855, and *PDE1C* PP785745, respective amino acid changes, and their genotype distribution in the T21, T21 + CHD, T21 + VSD, T21 + AVSD, T21-CHD and D21 groups.

Gene	Variant	Amino Acid Change	Genotype	Genotype frequencies					
				T21 (n = 1319)	T21 + CHD (n = 677)	T21 + VSD (n = 228)	T21 + AVSD (n = 151)	T21-CHD (n = 642)	D21 (n = 479)
*KIF7*	rs138354681	p.Gln834Arg	TT	0.986	0.973	0.939	0.974	1	0.994
			TC	0.014	0.027	0.061	0.026	0	0.006
*GJA1*	rs778110855	p.Ala311ValfsTer37	CC	0.992	0.984	0.952	1	1	1
			C/-	0.008	0.016	0.048	0	0	0
*PDE1C*	PP785745	p.Glu66Lys	GG	0.983	0.968	0.93	0.96	1	0.987
			GA	0.017	0.032	0.07	0.04	0	0.013

The probable pathogenicity of the variants was assessed using five programs, namely, PROVEAN, SIFT, MutationTaster, Polyphen-2 and CADD (Table 2). The *KIF7* rs138354681 was predicted to be ‘Disease Causing’ (Probability Score: 0.999) in MutationTaster, ‘Probably Damaging’ in Polyphen-2 (HumDiv score: 0.997; HumVar score: 0.986), ‘Deleterious’ in PROVEAN (Score: −3.381), ‘Intolerant’ in SIFT (Score: 0.02) and ‘Deleterious’ in CADD (Score: 24.1). In case of *GJA1* rs778110855, the predictions were ‘Disease Causing’ (Probability Score: 1) in MutationTaster, ‘Possibly Damaging’ in Polyphen-2 (HumDiv score: 0.945; HumVar score: 0.629), ‘Neutral’ in PROVEAN (Score: −1.665) and ‘Highly Deleterious’ (Score: 34) in CADD. The SIFT score and prediction for this indel variant were not available. The novel *PDE1C* PP785745 missense variant was predicted to be ‘Disease Causing’ (Probability Score: 0.999) in Mutation Taster, ‘Probably Damaging’ (HumDiv Score: 0.991) and ‘Possibly Damaging’ (HumVar Score: 0.76) in Polyphen-2, ‘Deleterious’ in PROVEAN (Score: −2.584), ‘Intolerant’ in SIFT (Score: 0.01) and ‘Highly Deleterious’ (Score: 30) in CADD.

**Table 2:** In-silico predictions along with score for potential pathogenicity of the studied variants using bioinformatic tools MutationTaster, Polyphen-2, PROVEAN, SIFT, CADD. HumDiv and HumVar scores for Polyphen-2 predictions are provided.

Gene	MutationTaster	Polyphen-2		PROVEAN	SIFT	CADD
Variant	(Model)	HumDiv	HumVar			
*KIF7* rs138354681	Disease Causing (0.999) (simple_aae model)	Probably Damaging (0.997)	Probably Damaging (0.986)	Deleterious (−3.381)	Intolerant (0.02)	Deleterious (24.1)
*GJA1* rs778110855	Disease Causing (1.0) (complex_aae model)	Possibly Damaging (0.945)	Possibly Damaging (0.629)	Neutral (−1.665)	N.A.	Highly Deleterious (34.0)
*PDE1C* PP785745	Disease Causing (0.999) (simple_aae model)	Probably Damaging (0.991)	Possibly Damaging (0.76)	Deleterious (−2.584)	Intolerant (0.01)	Highly Deleterious (30.0)

**Fig 1:**
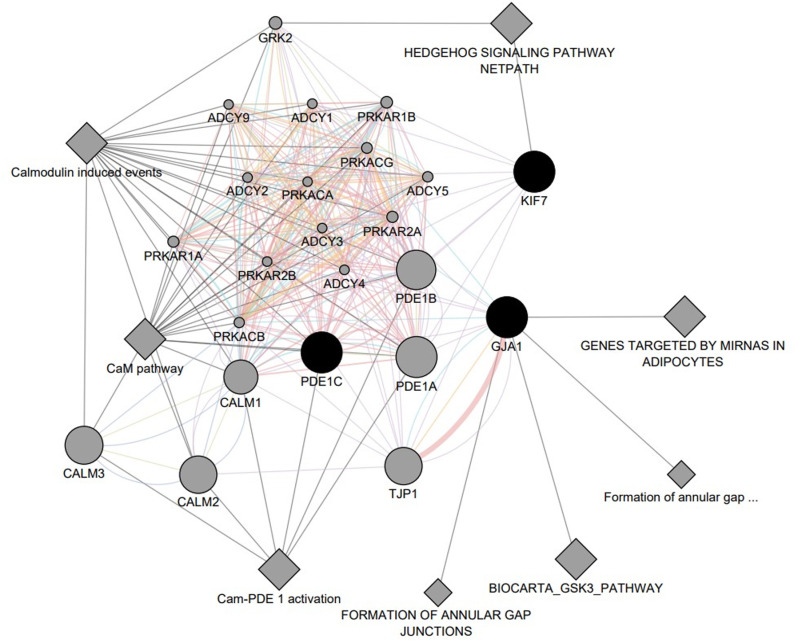
Interaction network among the studied genes KIF7, GJA1 and PDE1C obtained from the Cytoscape software.

Additionally, the support to our findings come from cytoscape and STRING analyses (Fig 1), which reveal potential functional interactions among *KIF7, GJA1, and PDE1C* genes with other known cardiac regulators. This analysis intuitively suggests any genetic variation that challenges optimal function of these three genes can potentially affect the other interacting genes that lead to CHD under the backdrop of T21 condition.

We were keen to observe if the simultaneous presence of these rare variants adds an additional level of complexity to the already vulnerable group like persons with T21 genetic condition. Among the 677 individuals with T21 + CHD, 31 individuals carried at least one variant. Within this subset of 31 individuals, the presence of only one of the variants was noted in 9 individuals (N1), the presence of any two variants was observed in 18 individuals (N2) and only 4 individuals (N3) carried all three variants. This stratification was done blinded without knowing the history of surgical intervention. We checked for the surgical intervention status of these 31 individuals from the epidemiological and clinical records following stratification. Interestingly, we found all 4 N3 individuals, required surgical intervention for the correction of their CHD as did 13 out of the 18 N2 individuals. Only 3 N1 individuals required surgical repair of the congenital cardiac defect, of which 2 were T21 + AVSD and 1 was T21 + VSD. In contrast, all the 4 N3 individuals, suffered from VSD. Among the 18 N2 individuals, 14 individuals suffered from VSD, of which 5 did not warrant surgical intervention while the other 9 did. Remaining 4 N2 individuals requiring surgical correction suffered from AVSD. We also observed the 9 N1 individuals carried the *PDE1C* novel variant only. Within the N2 group, we saw synergistic presence of *KIF7* and *PDE1C* variants in 11 individuals, that of *KIF7* and *GJA1* in 3 individuals and *GJA1* and *PDE1C* in 4 individuals. Among the 11 *KIF7* + *PDE1C* individuals, 8 required surgery. Two out of three *KIF7* + *GJA1* individuals required surgery and 3 out of 4 *GJA1* + *PDE1C* individuals required surgery. This distribution of genetic variants among the cases stratified by severity of CHD determined by need of surgical intervention or not is presented in the Table 3. The rarity of the three identified variants has been confirmed by comparing them with publicly available population databases such as gnomAD and 1000 Genomes, which have reported that these variants are either absent or extremely rare in global datasets. This suggests that the *KIF7*, *GJA1*, and *PDE1C* variants may be specific to the Indian Bengali population. All of our findings are novel as no other previous study has reported association of these three genetic variants with CHD in DS, as well as their cumulative effect on severity of CHD that necessitates surgical intervention.

**Table 3:** Distribution pattern of the number of variants present among the T21 + CHD individuals grouped according to their surgical intervention status.

31 T21 + CHD individuals carried at least one of the three studied variants							
9 T21 + CHD individuals carried a single variant (N1)			18 T21 + CHD individuals carried two variants (N2)			4 T21 + CHD individuals carried all three variants (N3)	
Surgery Not Required	Surgery Required		Surgery Not Required	Surgery Required		Surgery Not Required	Surgery Required
6 (0.67)	3 (0.33)		5 (0.28)	13 (0.72)		0	4 (1.0)
6 VSD	2 AVSD	1 VSD	5 VSD	4 AVSD	9 VSD	–	4 VSD

Statistical validation of our hypothesis regarding the cumulative effects of multiple variants on the expressivity (severity) of congenital heart defects in Down syndrome is supported by the polygenic risk score (PRS) values. The calculated beta values are *KIF7* = 0.8, *GJA1* = 1.2, and *PDE1C* = 0.5. A comparison revealed a significant difference in PRS values between controls and cases. The Shapiro-Wilk test supported the presence of significant non-normality in PRS scores (W = 0.31, p < 0.001). The Mann-Whitney U test result (U value = 36.0, p < 0.0001) suggests that case subjects who underwent surgical correction for congenital heart defects had a substantially higher PRS than those who did not. The surgery group had a median PRS score of 1.42 (interquartile range: 0.95–2.03), while the non-surgery group’s median PRS was 0.00 (interquartile range: 0.00–0.02). The effect size, determined by rank-biserial correlation, was r = 0.85, showing a strong relationship between PRS and surgery status. A Spearman’s correlation test also attested to the presence of a significant positive relationship between PRS scores and probability of surgery (ρ = 0.79, p < 0.001), and it indicated that higher PRS scores were likely to result in surgery.

**Fig 2:**
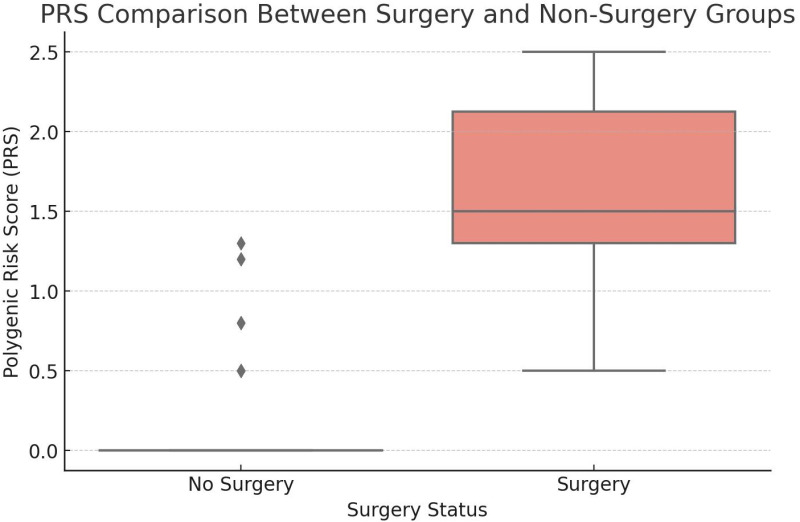
Polygenic Risk Score comparison between surgery and non-surgery groups of T21 + **CHD patients.**

The box plot (Fig 2) graphically emphasizes the difference between groups, with the surgery group having higher PRS values. The histogram with KDE (S2 Fig) indicates the right-skewed distribution of PRS scores, with the majority of people having low scores and a few outliers with high PRS values. In other words, an elevated cumulative genetic risk score may be a contributing factor in determining the need for surgery.

## Discussion

Congenital heart defects form a large subset of co-occurring conditions observed in the Down syndrome individuals and sometimes are life threatening. The type of CHD predominant among the DS cohort, has been seen to vary from one population to another. Studies on CHD in DS were initiated from the year 1991 as far published literatures are concerned, and through last three decades, many ethnicities and populations have been explored in this regard. We collected and studied metadata of all published reports on CHD categories among DS to compare our data with them and this is presented in Table 4. This metadata reveals AVSD is the most predominantly observed CHD in DS cohorts in as many as 11 studies encompassing 9 different populations, with some contradictory reports [20–30]. ASD was reported as most predominant DS associated CHD in 6 studies and VSD in 3 [31–39]. Four studies on Indian DS cohorts have reported VSD as the most common CHD in DS [40–43], and one study has reported it is ASD [44]. Our findings follow a similar trend to the previously reported Indian data, but are different from the global data, reporting VSD to be the most common type of CHD observed in our DS study cohort. This observation suggests the implication of population-specific factors that might contribute to this pattern of distribution of CHD sub-types among children with DS. However, we cannot rule out the possibility of increased mortality of the T21 + AVSD foetuses and neonates that lead to miscomprehension in scoring lower proportion of T21 + AVSD cases in our study cohort, when compared to the global data. This might be because of limited health care support for neonates with DS and poor diagnosis of T21 + AVSD cases among Indian public hospitals from where we mostly recruited our study cohort. In face of huge population burden and related health care demand with comparatively limited resource, DS is often considered a less prioritised group in Indian society. More incisive epidemiological survey is needed to ascertain this pattern.

**Table 4:** Distribution of CHD subtypes in Down syndrome cohorts across global populations and Indian subpopulations including the one reported in the current study.

Study Population/ Country	Study Period		Total participants in the study	No. of CHD cases observed	Distribution of CHD types	Reference	
**A.Global Population Distribution**							
Northern Ireland	1987-1989		81	34	**AVSD 38.2%** ASD 20.6% VSD 14.7% PDA 17.6% Others 8.9%	[30]	
Europe	1967-1993		5581	1431	**AVSD 38.5%** ASD 6.8% VSD 28% PDA 3.6% TOF 3.5% Others 19.6%	[25]	
USA	1983-1993		2894	1620	**AVSD 30.6%** ASD 11.2% VSD 11.4% PDA 6.1% TOF 1.7%	[29]	
USA	1989-1995		227	100	**AVSD 45%** ASD 8% VSD 35% PDA 7% TOF 4% Others 1%	[22]	
Sweden	1973-1980		219	104	**AVSD 41.3%** ASD 13.4% VSD 32.7% PDA 1.9% Others 4.4%	[23]	
Oman	1995-1998		54	54	AVSD 27.7% **ASD 33.3%** VSD 25.9% PDA 9.3% TOF 1.9% Others 1.9%	[38]	
Guatemala	1997-2003		349	189	AVSD 9.5% **VSD 27.5%** ASD 12.7% PDA 28.6% Others 21.7%	[39]	
USA	1992-2002		43463	15885	**ASD 48.8%**	[31]	
Oman	1996-2004		110	63	**AVSD 35%** VSD 9.5% ASD 25% PDA 9.5% Others 21%	[24]	
Libya	1995-2008		1193	537	**ASD 24%** AVSD 19% VSD 14% PDA 5% TOF 2% Others	[32]	
England	1985-2003		702	293	**AVSD 43.3%** ASD 17.7% VSD 31.7% TOF 5.1% Others 2.2%	[26]	
Europe	2000-2010		7044	3068	**ASD 40.6%**	[35]	
Korea	2005-2006		394	224	**ASD 53.5%**	[34]	
Jamaica	2007		53	42	**AVSD 54.8%** ASD 4.8% VSD 16.7% PDA 16.7% TOF 4.8% Others 2.2%	[27]	
France	1979-2008		728	323	**AVSD 30.3%** ASD 25.3% VSD 22.3% PDA 4.9% TOF 3.1% Others 14.1%	[28]	
Sweden	1992-2012		2588	1387	**AVSD 42%** ASD 16.1% VSD 22.1% PDA 5% TOF 3.3% Others 11.5%	[20]	
Iran	2012-2014		110	55	AVSD 12.73% **ASD 41.82%** VSD 14.54% PDA 20% TOF 10.91%	[36]	
Thailand	2009-2013		149	64	AVSD 20.3% ASD 20.3% **VSD 25%** PDA 21.9% TOF 6.25% Others 6%	[33]	
Norway	1994-2009		1251	724	**AVSD 35.1%** ASD 18.6% VSD 25.4% PDA 8.3% TOF 1.9% Others 10.7%	[21]	
Pakistan	2021-2022	123		123	AVSD 8.9% VSD 18.7% ASD 4.8% PDA 14.6% Complex AVSD 7.3% **Complex VSD 22.8%** Complex ASD 7.3% Complex TOF 8.1% Others 7.5%		[37]
**B.Indian Subpopulation Distribution**							
North India	2007-2008	50		25	AVSD 28% ASD 16% **VSD 48%** PDA 8%		[40]
South India	2005-2012	418		256	AVSD 27.3% ASD 12.5% **VSD 28.1%** PDA 16.8% TOF 3.9% Others 11.4%		[42]
South India	N.A.	50		27	AVSD 29.7% ASD 18.5% **VSD 40.7%** PDA 7.4% TOF 3.7%		[41]
North West India	2021-2022	57		35	ASD 20% **VSD 31.4%**		[43]
South India	2000-2020	610		356	AVSD 6.18% **ASD 30.34%** VSD 10.67% PDA 10.67% TOF 2.81% Combinations 33.15% Others 6.18%		[44]
**West Bengal, India**	**2018-2023**	**840**		**431**	**AVSD 22.3%** **ASD 25.7%** **VSD 33.6%** **PDA 10.9%** **TOF 1.2%** **Others 6.3%**		**Current Study**

The genetic basis for the incidence of CHD in a considerable proportion of DS individuals is likely multifactorial. The trisomic genetic background, in combination with allelic variants in various candidate genes and their interactions, might contribute to the manifestation of CHD in a subset of the DS population. Genetic variants linked to particular clinical phenotypes are often specific to populations, ethnic groups, and demography [45]. These population-specific variants can make the etiology of a disease more complex. Whole exome sequencing or NGS allows us to identify deep seated variants that might be overlooked by large population-based genome wide association studies. Earlier, candidate gene approaches have identified variants in genes *GATA5*, *FBLN2*, *CRELD1*, *FRZB*, *COL6A1*, and *COL6A2* [7]. Similar studies on the Indian population focused mainly on *CRELD1* [11,12] and folate metabolism pathway genes [8–10]. However, our current study, for the first time identifies potentially rare and novel pathogenic variants in the Indian DS cohort with CHD and we hypothesize that a genetic load threshold contributes to the increasing complexity and severity in clinical presentation of CHD that is reflected in the needs of surgical intervention. The synergistic effect of variants in two or more genes of cardiogenesis pathway or their regulators under T21 genetic backdrop cause a wider opening in the atrio-ventricular septum (in case of AVSD) or ventricular septum (in case of VSD), which is probably narrower among single variant carriers and subsequently closes without surgery, in post-natal developmental tenure. We observed most individuals (6/9) carrying a single variant underwent spontaneous closure of the septal defects. Among the 31 T21 + CHD individuals carrying the studied variants, 6 were T21 + AVSD and 25 were T21 + VSD. All the 6 T21 + AVSD individuals required surgical correction, of which 2 belonged to the N1 group and rest 4 belonged to the N2 group. Of the 25 T21 + VSD individuals, 7 carried one variant (N1), 14 carried two (N2), and 4 carried three (N3). Only 1 out of the 7 N1 T21 + VSD individuals required surgery, while 9 out of the 14 N2 T21 + VSD individuals and all 4 N3 T21 + VSD individuals needed surgical intervention. It is evident from our data, the *PDE1C* PP785745 variant, by itself does not contribute crucially to the requirement of surgery in the T21 + VSD cohort, but it clearly does so, when coupled with the *KIF7* rs138354681 or *GJA1* rs778110855 variants. Our analyses robustly support that a higher cumulative genetic risk, as quantified by polygenic risk scores (PRS), significantly contributes to the severity of congenital heart defects in Down syndrome, with surgical cases exhibiting markedly elevated PRS values compared to non-surgical cases. Moreover, the strong effect size (r = 0.85) and positive correlations (Spearman’s ρ = 0.79, p < 0.001) indicate that cumulative genetic risk assessment may serve as a valuable predictive marker for both disease expressivity and clinical decision-making.

**Fig 3:**
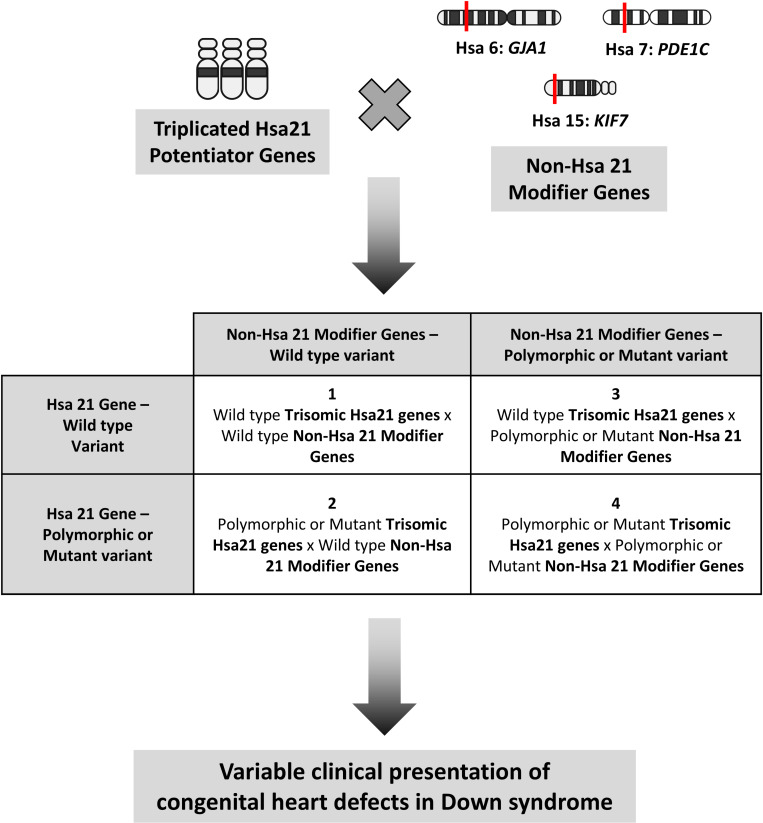
Graphical representation of the hypothesis regarding potential allelic interaction models among triplicated Hsa21 potentiator genes and disomic non-Hsa21 modifiers in manifestation of CHD in DS.

Interestingly, we did not find any common genetic variants that were previously reported as associated with CHD in DS and also from the genes located on Hsa21. Instead, our study revealed the presence of three distinct rare gene variants, *KIF7* rs138354681, *GJA1* rs778110855 and *PDE1C* PP785745 that have not been identified in any of previous studies to be associated with CHD in DS. The role of *KIF7* (Kinesin protein 7) gene in cilia-dependent developmental signalling is well studied [46]. It plays an important role in cardiovascular development [47]. *KIF7*, along with other proteins, plays an important role proteolytic processing of SHH signalling genes, and is associated with ventricular septum development [48]. *KIF7*^-/-^ mice were reported to show CHD [49]. Our study reports the presence of the rs138354681 missense variant to be present in 18 T21 + CHD individuals compared to none of the T21-CHD individuals. The presence of this potentially pathogenic rare variant might be associated in dysregulation of cilia-dependent *SHH* signalling during cardiac development, leading to development of CHD in DS. The *GJA1* gene codes the gap junction protein alpha 1, found in cardiomyocytes, and is associated with modulation of cardiac neural crest cell migration and thus, with cardiac development [50]. It has also been implicated in conotruncal heart defects [51]. Mutations of the *GJA1* gene have not been identified in any previous study as associated with CHDs (in DS and in any non-syndromic cases), but its role in cardiac development is reported [52]. In our study, the *GJA1* rs778110855 was found in 11 individuals from the T21 + CHD group. The frameshift deletion was not found in the T21-CHD group. The heterozygous ‘C/-’ genotype was also predicted to be pathogenic by three software and neutral by one. The SIFT program was not applicable in this case. We may infer that the delC variant might contribute to the increased incidence of CHD in DS from Indian population, probably by perturbing neural crest cell migration for cardiac septum development [53]. The gene *PDE1C* (Phosphodiesterase 1C) encodes a 3’5’-cyclic nucleotide phosphodiesterase enzyme, having a high expression level in cardiac tissue [54]. In mice, *PDE1C* protein shows progressively increasing levels through the phases of cardiac development [55]. Though the gene *PDE1C* or its mutations have not been reported in any previous studies as associated with CHD in DS or euploid populations, we have identified the presence of a novel missense variant PP785745, in 22 individuals of our Indian Bengali T21 + CHD cohort. The five in-silico prediction tools suggested the variant to be pathogenic. This novel variant has not been reported in any other population across the world, and we predict, the presence of this missense variant acts as a risk contributor for CHD in Indian DS population. The PDE1C protein interacts with LRP1 protein and PDGFRβ protein [56], contributing to their stability. Since *PDGFβ* signalling and *LRP1* have been associated with cardiac development as revealed in mice [57,58], it is intuitive that the novel PP785745 variant might lead to bizarre interactions among these proteins and subsequent dysregulated cardiogenic signalling. The network of interaction among these three genes have been represented in Fig 1, indicating an interplay of these genes among both structural (formation of gap junctions) and functional pathways (calmodulin induced events and hedgehog signalling pathways). This network strongly suggests that these genes interact among themselves functionally to lead correct regulatory signal for embryonic cardiogenesis. Deviation from any gene’s function owing to genetic variation may affect function of other genes that may result in CHD. It is intuitive that in euploid foetus (D21), the compromised function of these mutation bearing genes might be compensated by other genes that exert overlapping function (genetic redundancy), as it is known, a molecular back-up system exists for developmental mechanisms [59]. But, the T21 genetic background possibly imperils such cellular back-up system and thus, may exacerbate this anomaly many folds over D21 genetic condition. This is consistent with our findings, where none of the D21 carriers recorded any anomalous cardiac phenotype, whereas all of the T21 carriers did. The trisomy 21 genetic backdrop, in itself, thus acts as a potentiator, enhancing the implications of the variants. The non-occurrence of any genetic variants from Hsa21 candidates as risk of CHD is surprising, but possible. Best example of this possibility is the *CRELD1* variant which can increase the AVSD risk many folds under T21 condition though the gene is Hsa3 (3p25.1) candidate. Study on Ts65Dn mouse model revealed overexpression of the trisomic potentiator gene, *Jam2*, in interaction with disomic mutant genetic modifier *CRELD1* elevates the penetrance of CHD [60]. Similarly, *KIF7* (15q26.1), *GJA1* (6q22.31) and *PDE1C* (7p14.3) are modifiers of cardio-regulatory potentiator genes from Hsa21 (Fig 1) and their suboptimal functional interactions perturb the embryonic cardiogenesis. It is possible that Hsa21 potentiator candidate may have less genetic variations in Indian DS samples than other previously reported populations and for this reason we did not find Hsa21 candidate variants in our sample group. Alternatively, we may have missed to record their variants. Further deep genotyping could resolve this issue in future. To provide possible rationale for variations in clinical presentation of CHD in DS, we propose a model in which four genetic combinations (interactions) are possible (Fig 3): i) wild type triplicated Hsa21 with wild type non-Hsa21-modifiers, ii) mutated triplicated Hsa21 with wild type non-Hsa21 modifiers, iii) wild type triplicated Hsa21 genes with mutated non-Hsa21 modifiers, and iv) mutated triplicated Hsa21 genes with mutated non-Hsa21 modifiers. Among all four possibilities the first option is intuitively less penetrant condition and last option offers highest risk of CHD penetrance and severity. Our current finding fits with third possibility of this model. Additionally, presence of mutational load of more than one modifiers may add more complexity in genetic etiology of CHD in DS. All these possibilities are needed to be confirmed by careful experimental manipulation in preclinical models.

Careful observation of our findings shed light on common genetic regulations of atrio-ventricular septum and ventricular septum development as we found genetic variants that are common to both AVSD and VSD. It is possible some genetic modifiers with their allelic variants ultimately lead to AVSD or VSD in DS. Additionally, we observed that few subjects with two variants did not need surgery, that is, less severe manifestation of CHD. Conversely there were some subjects who exhibited only one identified variant but needed surgical intervention. It is possible that there are some genetic modifiers or regulators that complement partially the synergistic deleterious effects of two variants in some subjects. On the other hand, subjects with one variant may carry some mutations/alterations in other cardiogenic signalling loci that we failed to identify in genotyping. Further incisive studies are warrant to resolve this enigma.

Our study has limitations. Though we have taken opportunity to test a larger Indian DS cohort, we could not conduct WES for all subjects, owing to resource limitation. If, we could have accomplished WES for larger samples, many new variants of other cardio-regulatory genes and their genetic modifiers could be identified. So, implication of unidentified variants remained unaddressed in our study. We did not conduct GWAS study, which may have limited our approach to identify some other risk variants for CHD in DS from India. We focused solely on the genetic risk of CHD and presented the results in a straightforward manner, without considering potential confounding effects of maternal genotypes, maternal nutritional status (such as folate levels), or environmental factors. However, we cannot rule out their possible contributions to CHD phenotypes in DS. We could not demonstrate effects of over expression of Hsa21 genes on these three identified novel modifiers or perform a translational study due to non-availability of ethnicity-specific T21 cell lines in India. In relation to the novelty of our findings, we encountered challenges in interpreting the effects of the identified variants at the subcellular molecular level due to a lack of published literature. Nevertheless, our study has identified rare and novel variants associated with CHD in DS that contradict the hypothesis of Ramachandran et al. 2015 [3] who stated CHD in DS is underpinned by common variants. Our findings confirm that ethnically diverse populations may exhibit genetic variation that influences the clinical presentation of co-occurring conditions, such as CHD (or others), in DS. This study hints at the necessity of exploring other ethnic populations for the presence of such variants associated with CHD in DS.

## Conclusion

In conclusion, our study identifies the association of two rare and one novel potentially pathogenic variants with the incidence of CHD in individuals with DS and these variants had not previously been reported to linked to CHD in either trisomic or euploid populations. The finding suggests the existence of a threshold for these variants load, beyond which the synergistic contribution of the variants may further complicate the CHD phenotype, especially VSD in DS, necessitating surgical correction. These findings advance our understanding of the etiology of CHD in the trisomy 21 context and lay the groundwork for future studies to investigate the precise molecular mechanisms underlying this condition. We can infer that DS-associated co-occurring conditions, such as CHD, may arise from the triplication of Hsa21 genes across all populations. However, the effects of non-Hsa21 modifiers on the clinical presentation of CHD are population-specific. This hypothesis could also be extended to explain other comorbid conditions in DS. The findings of the present study enhance our understanding of the etiology of CHD in trisomy 21 and provide a foundation for future research to investigate population-specific modifiers and their alleles in other ethnic groups. Also, it lays down a foundation for future translational validation of the detected mutations in ethnicity-specific T21 cell lines. A high correlation between polygenic risk scores (PRS) and the probability of going for surgery was also found in our study. The effect size (r = 0.85) highlights the strength of this association, making PRS a potential predictive biomarker for surgical risk stratification. Subsequent research should investigate the biological underpinnings of this association and determine whether PRS can be utilized in clinical practice. This is consistent with past research indicating that patients with high PRS scores are more likely to develop diseases that necessitate surgery.

## Supporting information

S1 Fig Location of Hsa21q specific STR makers used for genotyping and confirmation trisomy 21 status of participating subjects. (TIF)

S2 Fig Histogram with kernel density estimation of Polygenic Risk Scores illustrating a right-skewed distribution, with most individuals displaying low scores and a few outliers exhibiting high values. (TIF)
