# Supplementary figures and images for: Rare and novel variant load threshold for KIF7, GJA1 and PDE1C genes elevates the risk of severity of congenital heart defects in Down syndrome
Source: PLoS One. 2025 Jun 26;20(6):e0326566. doi: 10.1371/journal.pone.0326566 (PMC12200707; doi:10.1371/journal.pone.0326566)

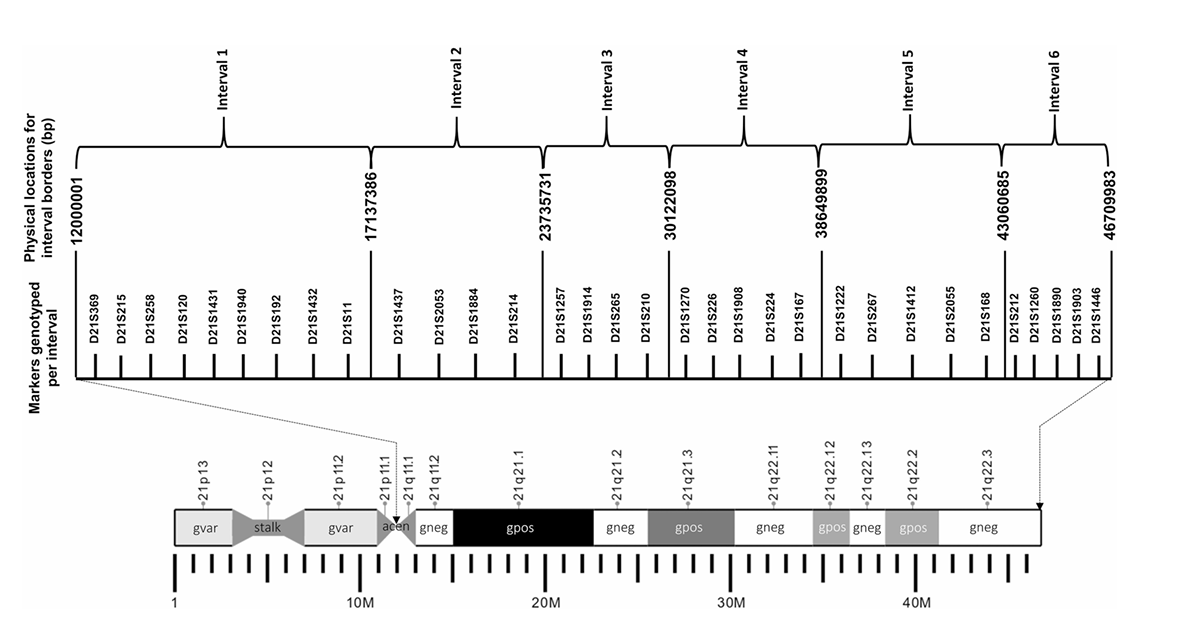

Supplement: S1 Fig — (TIF) [file pone.0326566.s001.tif]

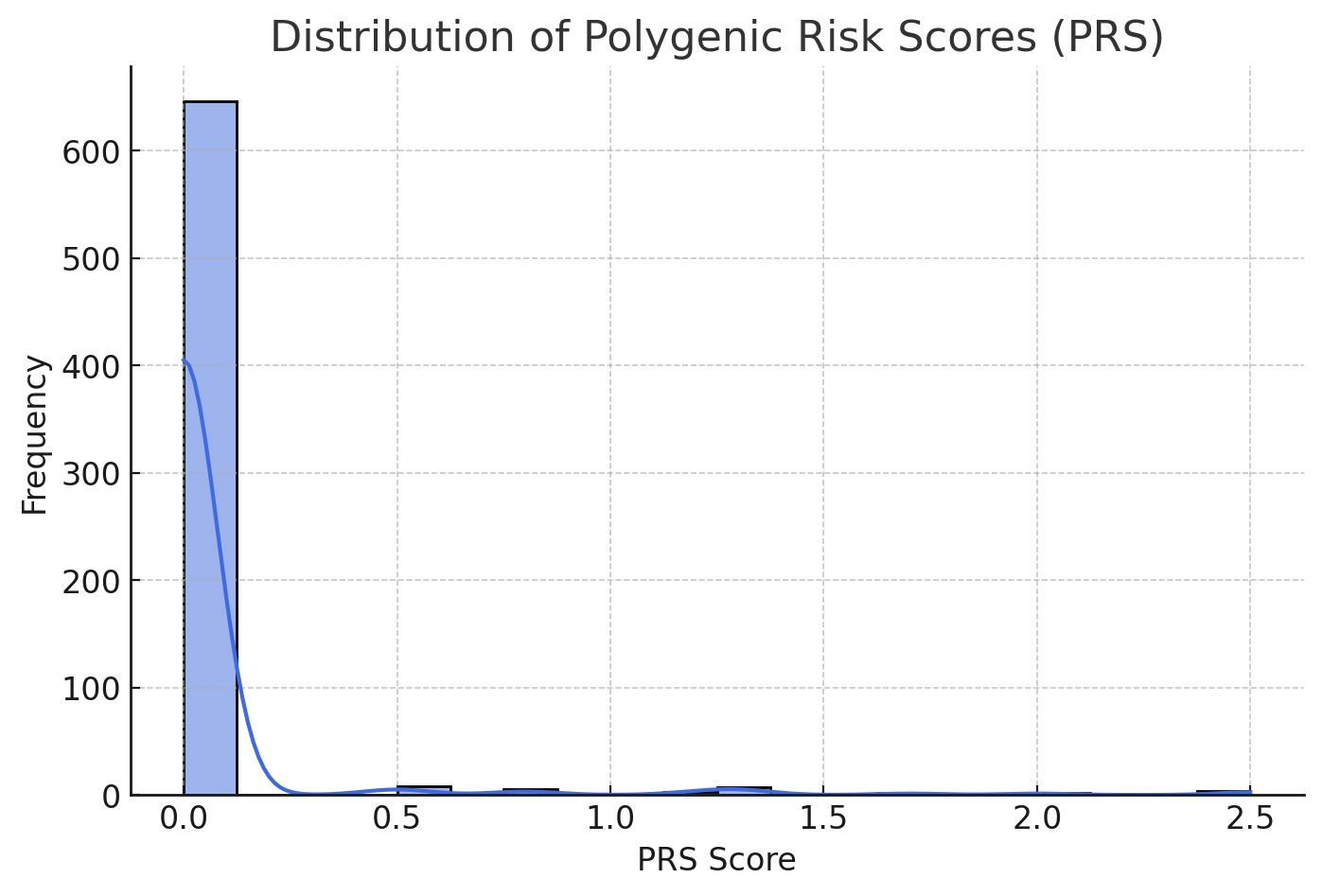

Supplement: S2 Fig — (TIF) [file pone.0326566.s002.tif]
